# Combined heat and power systems: economic and policy barriers to growth

**DOI:** 10.1186/1752-153X-6-S1-S3

**Published:** 2012-04-23

**Authors:** Adil Kalam, Abigail King, Ellen Moret, Upekha Weerasinghe

**Affiliations:** 1 Economics Department, University of Chicago, Chicago, IL, 60637, USA; 2 Ivring B. Harris Graduate School of Public Policy Studies, University of Chicago, Chicago, IL, 60637, USA

## Abstract

**Background:**

Combined Heat and Power (CHP) systems can provide a range of benefits to users with regards to efficiency, reliability, costs and environmental impact. Furthermore, increasing the amount of electricity generated by CHP systems in the United States has been identified as having significant potential for impressive economic and environmental outcomes on a national scale. Given the benefits from increasing the adoption of CHP technologies, there is value in improving our understanding of how desired increases in CHP adoption can be best achieved. These obstacles are currently understood to stem from regulatory as well as economic and technological barriers. In our research, we answer the following questions: Given the current policy and economic environment facing the CHP industry, what changes need to take place in this space in order for CHP systems to be competitive in the energy market?

**Methods:**

We focus our analysis primarily on Combined Heat and Power Systems that use natural gas turbines. Our analysis takes a two-pronged approach. We first conduct a statistical analysis of the impact of state policies on increases in electricity generated from CHP system. Second, we conduct a Cost-Benefit analysis to determine in which circumstances funding incentives are necessary to make CHP technologies cost-competitive.

**Results:**

Our policy analysis shows that regulatory improvements do not explain the growth in adoption of CHP technologies but hold the potential to encourage increases in electricity generated from CHP system in small-scale applications*.* Our Cost-Benefit analysis shows that CHP systems are only cost competitive in large-scale applications and that funding incentives would be necessary to make CHP technology cost-competitive in small-scale applications.

**Conclusion:**

From the synthesis of these analyses we conclude that because large-scale applications of natural gas turbines are already cost-competitive, policy initiatives aimed at a CHP market dominated primarily by large-scale (and therefore already cost-competitive) systems have not been effectively directed. Our recommendation is that for CHP technologies using natural gas turbines, policy focuses should be on increasing CHP growth in small-scale systems. This result can be best achieved through redirection of state and federal incentives, research and development, adoption of smart grid technology, and outreach and education.

## Background

### New era of energy production

The United States faces daunting pressures on the energy market. In the past decade, demand for electricity has increased, as have retail prices and fuel costs for electricity production. In the United States, Americans consume over three times as much electricity per year than they did 50 years ago. In 2006, electricity consumption per capita was 13,583 kWh per year [1]. And while the United States consumes nearly 25 percent of the world's energy, our population comprises only 5 percent of the world's population. Other issues affecting this marketplace are constraints on traditional electricity supply and delivery, global competition, climate change concerns, a failing grid infrastructure, and security issues.

### Growing pressures

• Electricity consumption has risen by 14 percent in the past ten years, going from approximately 8.9 billion kWh per day in 1998 to approximately 10.17 billion kWh per day in 2008.

• As demand for electricity increases, so have average prices per kWh, exerting increasing pressure on the U.S. energy situation. The residential sector saw prices rise from 8.3 cents per kWh in 1998 to 11.4 cents per kWh in 2008.

• As fuel costs for generating electricity have risen in the past decade, so have end use prices.

The picture is clear; the U.S. needs affordable solutions to combat increasing cost and demand pressure in electricity markets. Energy efficiency is understood to be the cornerstone of improving our future energy portfolio. Installing energy efficient technologies like commercial and industrial CHP are cost-negative. Combined Heat and Power technology is one of the most appealing energy efficiency measures available to us today; it can lower overall energy demand, reduce reliance on fuel for generations, increase the competitiveness of businesses, cut green-house gas emissions, and reduce the pressure for electricity grid infrastructure improvements. Combined Heat and Power, or CHP, is an immediately employable solution that can address the growing constraints on America’s energy future.

### The CHP process

Combined heat and power describes any system that simultaneously or sequentially generates electricity and recovers and re-uses the thermal energy byproduct of this process. CHP systems have huge energy efficiency improvements because they produce two forms of useful energy – heat and electricity, from a single fuel source. CHP extracts more useful energy from one fuel source than do the combination of processes that occur at traditional power plants that produce electricity and separate facilities that produce heat [2]. In comparison with a standard power plant, which operates at about 45 percent efficiency, a CHP facility typically operates at 80 percent efficiency.

CHP facilities extract this energy through two main types of power cycles known as topping and bottoming cycles. The topping cycle, also known as a combined cycle, is the most widely used and applied technology. A topping cycle system uses fuel to power the primary process of generating electrical power. Then the excess heat from this process is harvested and used directly to heat air or water, or as an energy source for heat-driven cooling systems. A bottoming cycle uses the primary fuel source to drive a heating mechanism. The excess heat from this process is then used to generate electricity for on-site use or to sell back to the electrical grid.

As the U.S. electricity grid becomes more focused on clean, renewable and efficient energy and moves away from a focus on centralized power plants, on-site power generation, known as Distributed Generation, is garnering increasing attention. Many types of CHP applications are forms of Distributed Generation. Typically, smaller-scale CHP systems produce a portion of the electricity needed by a facility some or all of the time on-site, with the balance of electric needs satisfied by purchase from the grid. CHP as a form of distributed generation increases efficiency in two important ways. First, the system itself recovers waste heat to generate more KWH per unit of fuel. Second, generating electricity on-site reduces the amount of energy lost in transmitting electricity.

### Modern history of CHP

Despite widespread use of CHP technology in the early 1900s, the technology’s share of the energy portfolio fell to 4 percent by 1978 [2]. As technology became more reliable and cost-effective, U.S. grid infrastructure transitioned to centralized utility generators and CHP was abandoned in favor of more convenient purchased electricity. But with the introduction of the Public Utilities Regulatory Policy Act of 1978 (PURPA) interest in CHP was renewed. PURPA included measures to promote CHP by offering incentives to utilities that purchased a portion of their power from distributed generation systems [3]. In the 1980s CHP grew rapidly at large industrial facilities with significant on-site heat and electricity demands. By the late 1990s the federal government realized that distributed generation facilities, particularly CHP systems, were a cost-effective way to meet rising energy needs. As a result, the U.S. Environmental Protection Agency and the Department of Energy have singled out CHP as an object of funding and attention and committed to increasing CHP capacity to 92GW by 2010. As of 2007, CHP facilities have been installed at 3,364 sites and have increased in total generating capacity from 46GW in 1998 to 85GW as of 2007 [3].

### Growing CHP

Combined Heat and Power is a proven, well established technology with a long history in the U.S. Installed capacity has increased from less than 10 GW in 1980 to 85 GW in 2006. But as a percentage of the overall power grid, this technology has not made substantial gains. CHP currently accounts for approximately 6.9 percent of total United States electricity generating capacity (MW) [4] and 7.9 percent of the U.S. electricity generation (MWh) [5]. The greatest portion of current installed CHP capacity is in the industrial sector and is the segment with the greatest potential for growth. Other areas for growth in CHP capacity include district energy systems, in which a central plant distributes steam to a network of locations, and small-scale systems in individual buildings. Advocates for energy efficiency have set a goal of increasing CHP capacity to 20 percent by 2030. Substantial additions like these to the electricity grid over the next twenty years will be influenced most heavily by two main factors: decreasing production costs and increasing favorable policies.

The biggest challenges facing the CHP industry are due to unfavorable state energy policies. On the federal level, policies that encourage the adoption of CHP are present, but not substantial. Improvements in federal policies should come in the form of streamlining depreciation schedules that are currently inconsistent across technologies, increasing Research and Development initiatives, and expanding investment and production tax credits for CHP technology.

Many state level policies are outdated relics from the age of centralized and inefficient utilities with great influence over the electricity grid. Some state energy policies require confusing and inconsistent permitting, lack interconnection standards, charge fees such as backup rates, standby rates and exit fees, enforce non-output based emission standards, couple utility revenues with electricity sales, and lack Renewable Portfolio Standards that include CHP. Our subsequent analysis will focus on the effect of these policies on CHP growth on a state-by-state basis.

### CHP advantages

Combined Heat and Power systems can provide a range of benefits to users with regards to efficiency, reliability, costs and environmental impact.

#### Reduced energy consumption through increased efficiency [5]

The primary benefit to using CHP systems is the potential for an increase in efficiency in both heat and electricity production [6]. The majority of fossil fueled power plants lose over two thirds of its energy in wasted thermal energy [7]. CHP systems generally can increase operating efficiency (in both electricity and thermal generation) from 33 percent overall efficiency (the average of U.S. fossil fueled power plants) to over 75 percent overall efficiency and sometimes can achieve operating efficiency as high as 88 percent as in the case of ExxonMobil’s Beaumont Refinery which operates a 470 MW CHP system and requires “37 percent less fuel than typical onsite thermal generation and purchased electricity” [7].

CHP systems can increase both total system efficiency as well as effective electric efficiency. This means it increases both the efficiency of the CHP system as compared to a system producing heat and compared to conventional electricity production. It uses less fuel to produce both heat and electricity. Apart from the obvious resulting reductions in operating costs there is a significant increase in power reliability and environmental quality.

#### Reduced energy costs

The reduction in energy costs results from a number of different factors associated with CHP systems. Because of the reduced energy consumption (due to high efficiency of CHP systems) energy costs can be reduced. This applies to the actual users of the CHP systems and the consumers of electricity bought from users of CHP systems. In order to determine the reduction in energy costs it will be necessary to determine the costs of the CHP technology (installation, fuel, operation and maintenance) and compare the price of electricity provided that would be required to make CHP a worthwhile investment with the price of electricity in a conventional CHP system [8]. It can also be useful to compare the costs of CHP with the other costs of thermal energy if the CHP system is also providing thermal outputs.

Aside from the direct benefits from reduced energy costs, CHP systems can also provide benefits from offset capital costs and improved reliability. Conventional power and thermal energy systems require boilers, chillers and other major heating or cooling equipment to be replaced or updated, and if CHP systems are installed in place of the now unnecessary boilers and chillers, the capital costs of installation are offset.

Furthermore, as a result of the reliability benefits described in the next section, there are also economic benefits from improved reliability for CHP users because CHP systems offer the ability to use CHP as backup power and allow users to supply their own power when prices of electricity are very high [8]. Because CHP systems are often located onsite, they allow users to avoid transmissions and distribution losses usually associated with conventional off-site energy generation.

#### Improved power reliability

Some of the previously mentioned economic benefits occur as a result of the improved power reliability that CHP systems can offer. Since CHP systems can be strategically located at point of use, the facility is less reliant on the electrical grid and has less chance of losing power. CHP systems provide electric and thermal power to sites on a continuous basis, allowing sites to provide their own power and thermal needs when the electricity prices are very high [9]. Therefore best conditions for adoption of CHP are when electricity prices are high and fuel costs are low.

Additionally, CHP systems can be used instead of additional generators to provide back-up power to a facility during power outages.

#### Improved environmental quality

When considering the environmental benefits from CHP systems, once again we can see the effects of increased efficiency of CHP technology. Since CHP systems require less fuel to produce the same amount of energy that conventional power systems produce, they can provide the same output while combusting less fuel and producing less air pollutants [10]. Most importantly, this reduces greenhouse gas emissions (such as carbon dioxide), other air pollutants (nitrogen oxide and sulfur dioxide) and water consumption.

According to a recent study conducted by the Department of Energy, current generating capacity of U.S. CHP sites is now almost 9 percent (85GW) of total U.S. capacity [11]. With this capacity, in 2006, U.S. CHP sites produced more than 12 percent (506 billion kWh) of total annual U.S. power generation. Their analysis shows that an increase in generating capacity to 20 percent of total U.S. capacity by 2030 would have significant positive outcomes [12].

Reductions in energy consumption would be equivalent to nearly half the total energy currently consumed by U.S. households and related policies could generate $234 billion in new investments and create nearly 1 million new highly-skilled, technical jobs throughout the United States [12]. Reductions in CO_2_ emissions would be the equivalent of taking more than half of the current passenger vehicles in the U.S. off the road and would avoid over 60 percent of the projected increase in CO_2_ emissions between now and 2030 [12].

## Technology

One of CHP’s greatest advantages is its wide applicability and integration into residential, commercial and industrial processes. A variety of fuel options are available for CHP systems, and installations range from small- to large-scale facilities that span many sectors of the economy. The section below includes a discussion of natural gas turbines, the focus of this paper, followed by a table describing other CHP technologies that illustrate the breadth of technologies available for CHP adoption.

### Gas turbines

Historically, gas turbines were used for peaking capacity, but technological advancements have led to the utilization of gas turbines for baseload power and now make up forty percent of electrical market capacity additions. Much of the growth has been concentrated in large-scale facilities (< 50 MW) that use combined cycle technology that experience low capital costs and high efficiency [13]. Gas turbines are implemented in a variety of areas including oil recovery, chemicals, paper production, food processing, and universities. In a gas turbine, when air is compressed and ignited by fuel, the expansion of the heated combustion gases passes through both gas producing and power turbines, driving the compressor and the electric generator. Instead of wasting the resulting exhaust heat, the CHP system captures it, and uses it to heat water in the boiler producing high-pressure steam, which is put through the steam turbine producing more electricity [14].

Gas turbine sizes typically range from 500 kW to 250 MW. Industrial gas turbines range from 1 MW-250 MW, are heavier and less efficient than smaller gas turbines, and are best used for continuous generation of baseload power. They can include simple-cycle and combined cycle systems, both of which are suitable for CHP because of the high temperature exhaust produced during the process of energy generation. Simple cycle systems are less efficient because there is no recovery of heat in the exhaust gas, unlike the recovery process utilized in combined cycle systems [13]. Simple-cycle gas turbine CHP systems are most prevalent in smaller installations, typically less than 40 MW [13]. A combined cycle gas turbine is the most efficient commercial technology for central station power-only generation, with efficiencies approaching 60 percent lower heating value (LHV). A simple-cycle gas turbine reaches efficiencies of only 40 percent, but a gas turbine with CHP can achieve 70-80 percent overall efficiency [15]. Gas turbines for CHP systems are cost-effective in commercial or industrial applications with a generating capacity above 5 MW, and are often used for district energy systems because their high quality thermal output can be used for most medium pressure steam systems [13].

### Other CHP technologies

Although the focus of this paper does not include the technology types below, it is important to have a broad overview of technologies applicable to CHP development. Steam turbines are widely used in CHP systems because most of the electricity currently produced in the United States comes from steam turbines. Because the cost per kilowatt of a steam turbine CHP system is high due to its low power to heat ratio, it is most commonly used in medium-to-large scale industrial facilities. Benefits to CHP steam turbines include increased boiler efficiencies ranging from 70 to 85 percent, and the availability of a wide range of fuels (natural gas, coal, oils, municipal solid wastes, sludges). Microturbines, or small gas turbines, are more complex than simple-cycle gas turbines and are best used for distributed generation because of the flexibility in grid connection methods. Reciprocating engines are best used in CHP systems for commercial and institutional buildings that use space heating and have hot water requirements. Fuel cells currently experience high costs, but may become more cost-effective in the future as the technology matures.

### Size and location

CHP systems are particularly beneficial because of they have a wide range of capacities. This allows for a lot of flexibility in potential CHP applications, as CHP can be applied across a many different facility types and sizes. Outlined below are the three different system sizes (large scale, district energy, and small-scale system).

#### Large-scale

Gas turbines can be used in large-scale industrial facilities where combined-cycle CHP systems maximize power production to sell back to the grid. The electrical outputs of these facilities range in the hundreds of megawatts.

#### District energy

District energy systems include cities, campuses, hospitals, and other similar complexes and range from 5 to 50 MW [16].

#### Small-scale

The smallest scale facilities include residential settings, also referred to as micro-generation and mini-cogeneration. Micro-generation has a capacity of less than 5 kWe in a house or small business. Mini cogeneration facilities are usually more than 5 kWe and less than 500 kWe in a building or medium sized business. Small-scale CHP applications may include multifamily residential buildings, supermarkets, and hotels. A typical college or university campus might have a 5 MW simple-cycle gas turbine. Steam (or hot water) is produced in an unfired heat recovery steam generator and sent into a central thermal loop for campus space heating during winter months or to single-effect absorption chillers to provide cooling during the summer [17].

### Future technological developments

By the early 1980s, gas turbines had developed enough technologically in terms of their efficiency and reliability to become used in many different applications [18]. With efficiency ratings exceeding 70 percent, much of the development of CHP has been concentrated on using natural gas a fuel to power these turbines. However, with pushes in the renewable energy space and the emphasis on efficiency being extended, there have been developments in other areas, namely microturbines and fuel cells. Much of this is driven by the need to provide smaller CHP units that can be placed in buildings or homes as well by the need for more environmentally friendly technologies.

#### Microturbines

Microturbines, which are small electricity generators that burn gaseous and liquid fuels to create high-speed rotation that turns an electrical generator, began field-testing in 1997 [19]. These microturbines can be used in power-only generation or in combined heat and power systems, just as larger gas turbines are used. Within CHP applications, the waste heat from a microturbine is used to produce hot water, to either heat buildings, drive absorption cooling, and to supply other thermal energy needs. A major advantage of microturbines is their ability to operate on a variety of different input fuels – natural gas, sour gases (high sulfur, low Btu content), and liquid fuels such as gasoline, kerosene, and diesel fuel/distillate heating oil. This allows for the potential hedging of energy input costs, as the price of natural gas can fluctuate, which is illustrated in a later section.

#### Fuel cells

Fuel cell technology is another major area of development and has the potential to allow a small sized cell to power an entire home [20]. The advantage lies in that fuel cells produce electricity through a chemical reaction rather than by burning fuel, resulting in much lower emissions than its competitor technologies. The chemical reaction does, however, produce carbon dioxide, which is pollutant, but does so in much lower quantities. Furthermore, the higher efficiency of fuel cells allows for lower fuel usage, reduced noise pollution, and the lack of a centralized system/generation plants. As it stands now, fuel cell CHP systems are very expensive and focused on the premium power market with a need for the advanced benefits that fuel cells provide. Finally, the National Electric Code (NEC) and the National Fire Protection Association (NFPA) codes will apply to fuel cells used in residential applications; however, regulations concerning the connection of fuel cells with the home electrical system are still being developed.

#### Solar CHP

Another technology that is in development presents a very unique dynamic with combined heat and power systems. The potential for photovoltaic and solar thermal technologies has been presented through decades of research; however, these technologies are among the most expensive sources of renewable power [21]. With the development of concentrating solar PV-thermal hybrid technologies, otherwise referred to as Solar CHP, could potentially reduce the cost of solar power by making use of the electrical and thermal energy captured by a collector while reducing the materials cost through concentration. The system makes use of thermal energy to offset conventional fuel consumption. There are already patented solar CHP systems in the development phase, looking for opportunities to commercialize. The potential for solar CHP is emerging, and while it does not have the clout of microturbines and fuel cells, it does have the investment and research fueling its advancement.

## Statistical analysis of state policies

### Policy environment

The policy environment in which the CHP industry operates is multi-faceted; energy policy is unique and complicated by nature of the electricity grid infrastructure. The Federal Energy Regulatory Commission has authority over inter-, but not intra-state electricity sales, which means that state electricity policy has an enormous effect on CHP outcomes. Current policies effecting CHP can be broadly split into two categories. Financial policies such as tax credits to encourage private investment in CHP and government funding of Research and Development projects exist on both the state and federal level, but are more influential on a federal scale. Regulatory policies and institutional systems demonstrate huge variability across states and are purported to be one of the most important factors in the adoption of CHP. Our analysis section will further explore this notion in hopes of identifying the most important obstacles facing the industry.

### Funding and financial incentives – federal level

All federal level policy impacting the CHP marketplace comes in the form of incentives. While some non-fiscal federal policies do impact CHP indirectly through electricity grid regulations through FERC and other interstate commerce issues, the policies that are most influential to CHP development are the result of direct funding and incentives. The Federal Investment Tax Credit and Production Credit, Federal Tax Depreciation Schedules, Research and Development funding, and other initiatives like the CHP Partnership comprise the national policy space facing Combined Heat and Power systems. Natural Gas Turbine CHP systems are able to take advantage of most national funding resources, detailed here.

#### Federal CHP investment tax credit

The Energy Improvement and Extension Act of 2008 created a ten percent investment tax credit (ITC) for the costs of the first 15 MW of CHP properties. To qualify for the tax credit, the CHP system must produce at least 20% of its useful energy as electricity and twenty percent in the form of useful thermal energy. The ITC is only extended to systems smaller than 50MW and to natural gas turbine systems (or other non-biomass fueled systems) that achieve at least sixty percent efficiency. The ITC may be used to offset the alternative minimum tax, and the CHP system must be operational in the year in which the credit is first taken. The American Recover and Reinvestment Act of 2009 extended the scope of the Federal CHP Investment Tax Credit by extending the option of a grant of equal value in lieu of a tax liability reduction.

#### Qualified Energy Conservation Bonds (QECB)

The 2007 Energy Independence and Security Act created a funding mechanism similar to Clean Renewable Energy Bonds, and similar to other Production Tax Credits, which awards bonds in the form of tax credits instead of paying out interest. The system operates by authorizing state and local governments to issue QECBs and funds up to $800 million through the IRS. The 2009 stimulus increased the bonding authority by $2.4 billion.

#### Federal bonus depreciation schedules

Businesses may recover investments in certain property through depreciation deductions. This policy establishes a set of class lives for various types of property, ranging from three to 50 years, over which the property may be depreciated. The bonus depreciation schedule allows businesses to take half of the depreciation value of CHP property off of their tax liability for the first year, and the remaining half over the course of the next four years.

#### Federal research and development grants

Two programs currently exist on the federal level to directly stimulate innovation in the CHP sector. The Department of Energy Climate Change Technology Program provides $3 million to encourage research, development, demonstration and deployment of technology to reduce greenhouse gas emissions. The DOE’s Inventions and Innovations Program offers financial and technical support through competitive grants for research and development of innovative, energy-saving inventions.

#### Other grants, rebates and loans

The Federal Government provides funds for a variety of competitive grant and loan programs for renewable energy and energy efficiency programs, for which CHP systems may be eligible. These include:

• The Rural Energy for America Program, for which agricultural producers are eligible to receive grants for 25 percent of costs or loans for 75 percent of costs.

• The Advanced Power Systems Tech Program, part of the 2005 Energy Policy Act, offers a rebate of 1.8 cents per kWh of electricity generation up to the first 10 million kWh per year.

• Energy Efficiency and Conservation Block Grant provides $3.2 billion in formula and competitive grants to local and state governments for energy efficiency improvements in order to reduce energy use and fossil fuel emissions.

• DOE Energy Efficiency/Renewable Energy Loan Guarantees under the 2005 Energy Policy Act offers $10 billion for energy efficiency, renewable energy and advanced transmission and distribution projects for up to 100 percent of the amount of a loan that funds up to 80 percent of total project costs.

• Energy Efficient Commercial Buildings Tax Deduction.

• Energy Opportunities Program: Rebate.

• State Energy Program: Provides grants to states.

• DOE Grant Program: Deployment of CHP Systems, District Energy Systems, Waste Energy Recovery Systems, and Efficient Industrial Equipment.

• Combined Heat and Power Systems Technology Development Demonstration.

• Waste Energy Recovery Registry and Grant Program.

#### State financial incentives

Hundreds of state programs exist in the form of grants, rebates, loans, loan guarantees, and tax incentives.

#### CHP partnership (EPA)

In 2001, the Department of Energy (DOE) and U.S. Environmental Protection Agency (EPA) have collaborated to establish the CHP Partnership, a voluntary program aimed at encouraging CHP growth in the United States. The partnership fosters relationships between interested stakeholders including industry, state, and local governments, and promotes energy efficient CHP technologies [22]. Regional Application Centers have been established through the Partnership that target CHP development by region, providing analysis and information for those interested in CHP systems. This Partnership provides education and outreach activities to help promote growth in the CHP sector.

### Regulatory and institutional barriers

Financial incentives are one aspect of the policy arena affecting CHP development. Other limits to CHP adoption result from regulatory and institutional barriers located mostly at the state-level of government [22]. These regulations can encourage or inhibit adoption of CHP facilities. Because CHP and other forms of distributed generation operate under a broad framework of energy production, distribution and regulation, changes in this framework can influence the extent to which CHP is developed [23]. Described below is a comprehensive list of regulatory barriers, and suggestions as to how state regulations might be streamlined to appropriately incentivize CHP application.

#### Interconnection standards

Interconnection is the ability of a nonutility generator to operate while connected to the electric transmission/distribution system. Most CHP facilities must interconnect with the electric grid for backup power, in case the facility cannot generate enough electricity on its own, or in the event that it experiences an outage; as well as to sell back any excess power it produces. While CHP systems may improve the reliability of the grid by reducing grid congestion, many states do not facilitate connection to the grid. There is a general lack of uniformity in processes and fees, and the enforcement of current standards makes it difficult for manufacturers to design and/or produce modular packages that may be sold in large quantities [24]. This reduces the incentive for CHP implementation, particularly for small-scale systems that must predict the costs and requirements for access to the grid. There is also a problem with jurisdiction, which is split between the Federal Energy Regulatory Commission (FERC) and the states’ utility regulatory body. Currently, each utility and service territory establishes its own interconnection rules. Models and procedures have been developed by federal agencies, but none are mandatory or enforceable. The Institute of Electrical and Electronic Engineers (IEEE) developed IEEE 1547 *Standard for Distributed Resources Interconnected with Electric Power Systems*, which outlines procedures and requirements for the testing, operation, safety and maintenance of the interconnection of distributed resources. The Energy Policy Act of 2005 required state commissions to consider the standards proposed by IEEE but did not mandate adoption of the standards [24].

Fifteen states have adopted interconnection standards that are favorable to distributed generation. The standards establish clear guidelines that streamline the process, as well as provide technical requirements that reduce interconnection and cost uncertainty [25].

#### Utility rate structures/decoupling

Many utility rate structures provide utilities with a disincentive to promote energy efficiency, including CHP systems. By linking utility revenues with number of kWh sold, utilities benefit from maximum electrical output [26]. It inhibits companies from supporting more efficient energy resources. Decoupling programs to disassociate revenues from sales force utilities to appropriately value efficiency and saved costs, making CHP implementation easier. This better aligns utilities’ profit motives with the goal of providing power at the least cost to consumers [27]. Other examples of misaligned rate incentives include high standby charges for CHP systems, making CHP rates too costly to compete with baseload power; penalties for using electricity from the grid during unplanned outages; lower rates for companies considering CHP, making CHP less attractive; and costly exit fees. All of these additional costs can make CHP installations prohibitively expensive for smaller installations, and perhaps for larger facilities as well [28]. Currently, eight states have adopted standards that value the true costs and benefits of distributed generation, including CHP.

#### Renewable Portfolio Standards (RPS)

Renewable Portfolio Standards are state-adopted policies that require utility providers to generate a percentage of their energy generation from renewable energy sources by a particular date. Renewable Portfolio Standards are established to promote the growth of renewable energy as part of a state’s overall energy portfolio. Currently, 24 states have RPS in place, although the requirements and goals differ by state. Fourteen states have RPSs that include CHP as an eligible technology [29]. Adopting RPS standards that include CHP would encourage utilities to make the process of CHP implementation easier for facilities of all sizes because growth in the CHP sector would be essentially mandated by the state.

#### Output-based regulations and permitting policies

Output-based emissions regulations, as air pollution control mechanisms, limit emissions based on emissions output, rather than input. Traditionally, states have placed environmental regulations on the input of emissions [30], the fuel that goes into a power plant or energy-using facility. Because CHP systems may use more input fuel than conventional systems, input regulations discourage CHP implementation. Output-based regulations, however, encourage the adoption of CHP because it takes into account energy efficiency. CHP systems use less fuel per unit of output than a conventional system, and the energy savings would be incorporated into output-based regulations [31]. The energy savings gained from a CHP system is shown in Figure 5. Currently, twelve states have adopted output-based regulations standards that reflect efficiency improvements as pollution prevention.

An example of a federal environmental permitting barrier is the Clean Air Act’s New Source Review (NSR), which requires large point-source polluters to install pollution control components. These components can sometimes interfere with CHP adoption, further reducing incentives to install CHP [26].

#### Electricity restructuring

Electricity restructuring refers to the deregulation of the electricity market, or the movement from the traditionally regulated monopoly system towards competitive markets. Deregulation changes the landscape under which CHP systems (and the power industry as a whole) operate. Beginning in the early 1990s, federal initiatives, including PURPA and the Energy Policy Act of 1992 began encouraging competition in the electricity market [32]. State regulators began investigating restructuring around that same time, and there are currently fourteen states that have restructured the electric industry to make utilities more competitive. Eight states have suspended restructuring activities[33]. Although some basic features of restructuring policies are consistent across states, details of the legislation vary greatly. Competition in the electricity market will provide opportunity for CHP adoption, although variations between states make the effect difficult to predict.

#### Net metering

Net metering allows utility customers to offset all or part of their electricity needs by producing their own electricity, and selling excess power back at a one-to-one credit per kWh[29]. Net metering encourages investment in renewable energy and energy efficiency technologies, and may improve transmission grid reliability if consumers are producing electricity during peak periods [30]. In conjunction with interconnection standards, net metering is thought to help encourage CHP expansion by lowering costs to enter the electricity market. As of August 2009, forty-two states have adopted net metering policies. Of those, fifteen states allow CHP as an eligible technology for net metering [34].

### Methodology

Strong supporters of CHP growth exist across the nation in all levels of government, private sector and non-profit groups. In the 1990s the U.S. EPA and DOE took on the growth of the CHP sector as a goal at both agencies, which resulted in the creation of the EPA CHP Partnership, eight Regional CHP Application Centers, the DOE CHP Applications Program and increased research at DOE National Laboratories. Trade organizations in the private sector like the United States Clean Heat and Power Association, interest groups like the Northeast Combined Heat and Power Initiative, and advocacy organizations like the American Council for an Energy Efficient Economy and the Regulatory Assistance Project all vary in their approaches to advocating for CHP adoption, but they all share the goal of removing barriers to CHP development.

Across the spectrum of these groups and the associated literature is the assertion that unfavorable regulations at the state level are the most numerous and influential barriers facing the advancement of CHP systems. Despite the breadth of material regarding the removal of inconsistencies in standards, fees, permitting procedures and other state regulations detailed above, there is a lack of statistical analysis of the direct effect of policies on the growth of the CHP industry. Here, we attempt to add to the current knowledge of the most important challenges facing the Combined Heat and Power Industry. Can increases and decreases in the amount of electricity generated from CHP systems on a state-by-state basis from 1997 to 2007 be explained by the introduction or elimination of favorable policies within that time period?

We performed a logistic regression to assess the significance of seven categorical variables and one continuous variable against the dependent variable of the change in the share of CHP generating capacity. Results were evaluated with T-tests, using P-Values and Adjusted R-Squared statistics.

The sources for the dataset include the DOE’s Energy Information Administration (EIA), EPA, Oak Ridge National Laboratory (ORNL) and the Database of State Incentives for Renewables & Efficiency (DSIRE), which is funded by the DOE’s Office of Energy Efficiency and Renewable Energy (EERE) and operated by the National Renewable Energy Laboratory (NREL).

The dependent variable is the percent change of total electricity production between 1997 and 2007 for each state. Independent variables included the following categorical variables: interconnection standards, renewable portfolio standards, output-based regulations, electricity restructuring, net metering, utility standby rates, and technical potential. We also used the change in average retail price of electricity in cents per kWh from 1997-2007 as an independent variable to determine if the price of electricity had an effect on CHP implementation. See additional file 1 for additional information on methodology, including dataset sources, variable definitions and statistical output results.

### Results

The analysis indicates that at a 90 percent confidence interval, changes in the market share of CHP systems cannot be fully explained by state policy conditions facing the industry. Of the five states that have seen the most growth in CHP capacity, only Delaware has more than one favorable CHP regulation in place.

### Discussion

The results imply that there are other, possibly more significant factors that explain the share of electricity generation from CHP systems and the share of electricity producers over the past ten years. The analysis does not quantify financial incentives, and they may play a more significant role than policies. As the Cost-Benefit Analysis (Analysis 2) shows, CHP is already competitive in large-scale systems, meaning that the growth would not be due to favorable policies, but rather the cost-effectiveness of CHP systems. This argument is strengthened by the fact that during the years covered in this analysis, much of the growth was concentrated in large-scale facilities.

Although the results indicate that state regulatory and institutional policies do not affect CHP capacity growth, it does not necessarily suggest that the policies are completely ineffective. The analysis covered CHP growth during the years 1997-2007, which were probably a result of PURPA and much of the growth occurred in large-scale facilities. The trend in CHP and electricity generation in general is moving towards distributed generation, as opposed to the traditional baseload power configuration that exists today. State regulations that favor CHP would make smaller installations more cost effective, and may have a more significant effect on CHP growth in the future.

#### Opportunities for further analysis

A statistical analysis of the relationships between favorable CHP policies and growth rates of the sector could be improved and expanded by creating a larger database of policies that could show dynamic policy variables. For the purposes of our study, these variables are static indicators that occurred during the time period of the study. If changes in policy were included from before the time horizon, or were examined on a yearly basis, we might see more nuanced results. Another opportunity for further analysis is to examine the potential time lag between CHP-related policy implementation and CHP nameplate capacity changes. The scope of this study did not include financial incentives offered by states; a more sophisticated model that included these indicators could shed further light on the relationships in question.

## CHP cost-benefit analysis

### Economic overview

What are the specific costs and benefits associated with CHP? How have they affected the adoption of CHP technologies? Here we examine the impact of current CHP policies and financial incentives on the costs of CHP systems.

By looking at the broader energy industry, its future outlook can provide information to treat considerations of combined heat and power potential. To begin, EIA projections for future energy prices across sectors provide a broad overview of the energy industry’s outlook [35].

EIA calculations have shown that U.S. energy use per capita has been relatively stable since 1980 at 310 to 360 million Btu per person. However, during periods of high energy prices, namely oil prices, energy consumption per capita has treaded towards the lower end of the spectrum noted above.

The EIA projects that with oil prices expected to remain high throughout the period. And with the recent policy initiatives to increase energy efficiency, energy use per capita will drop below 310 million Btu in 2020 and continue to decline at a slow rate through 2030. Furthermore, the price of electricity per kWh has been steadily rising and will continue to do so through 2010. The following graphs from the EIA illustrate these projections.

Primary energy consumption is projected to grow by 0.5 percent per year from 2007 to 2030, with an annual demand for renewable fuels growing at the fastest rate. Biomass consumption increases by 4.4 percent per year on average over the same time period and makes up 22 percent of total marketed renewable energy consumption in 2030, as opposed to 10 percent in 2007. Also, natural gas and liquids for heating shows limited growth, with commercial natural gas use grows by 0.6 percent per year on average from 2007 to 2030 in the reference case used by the EIA, including more use of CHP in the later years. Commercial natural gas use in 2030 varies slightly in response to changing economic assumptions, from 3.4 quadrillion Btu to 3.7 quadrillion Btu, shown in the graphs below. Heat produced by fossil fuel fired generators in CHP applications can be used for water and space heating, increasing efficiency of the technology. However, the increase in natural gas used in current CHP systems in the commercial sector raises total natural gas consumption. On the other hand, the additional natural gas used for CHP systems in the commercial sector raises total natural gas consumption in the reference case and offsets some of the reductions in energy costs that result from efficiency gains in end-use equipment and building shells in the high technology and best technology cases.

A study conducted by McKinsey & Company in 2007 on reducing U.S. greenhouse gas emissions pointed that CHP can deliver CO_2_ reductions at a negative marginal cost for both the commercial and industrial sectors. As it stands, there are limiting factors to the implementation of CHP through an economic and regulatory lens. The electrical rate structure in place that links utility revenues to the number of kW hours sold are a barrier for major utility companies to implement on-site CHP generation. Also, the rate structure in place that would typically recover the majority of the cost of service fixed charges is not accounted for when applied to CHP, therefore reducing its cost saving potential [37].

Beyond that, the economic viability of CHP for many customers would require the integration of CHP systems into the utility grid for backup and additional power needs. The interconnection issues that presently exist revolve around the lack of uniformity in application processes as well as the difficulty in designing systems when standards are not enforced. In 2003, the Institute of Electrical and Electronics Engineers (IEEE) approved IEEE 1547 Standard for Interconnecting Distributed Resources with Electrical Power Systems, which was renewed in 2008. This detailed the requirements relevant to the performance and operation of the interconnection of distributed resources. Based on this, the Energy Policy Act of 2005 calls for state commissions to consider these standards but does not require them to adopt them. CHP’s economic viability becomes comprised without the ability to integrate and interconnect with the rest of the utility grid.

Also, given the current economic climate, the near future does not hold any significant increases in the Federal Funds Interest rate, which is at a low level compared to years past [37].

Finally, in regards to financing, the decision to invest in a CHP project is based on a projection of cash flows over time. This estimates the revenues and cost over the life of a project including factors such as energy prices, financing costs, depreciation, and tax considerations. The strongest indicator of a project’s financial strength is the ability of the project to make the debt payments. This is often calculated through the debt coverage ratio (operating income to debt requirements). Government bonds can achieve this with lower interest rate on their debt. This is in opposition to project finance, which requires a higher interest rate as well as an internal rate of return on equity [38].

In this analysis we continue to try and identify, through a cost benefit analysis, the funding opportunities with highest potential to increase optimal adoption of CHP technologies. As we have already indicated, there is a range of federal and state incentives aimed, through various measures, at encouraging the adoption of CHP technology.

In order to determine the effectiveness of some of these incentives, our previous analysis focused on determining the impact of non-funding related state incentives on increases in CHP applications Nevertheless, as the previous analysis has shown, regulatory incentives have not increased the adoption of CHP technologies in applications throughout the United States.

Given that previous analysis to determine whether there is a significant *effect* of incentives on increasing CHP adoption, this analysis attempts to determine whether there is a significant *need* for incentives in order to allow CHP technologies to be adopted in a competitive market. In other words, this study attempts to discover whether CHP technologies can be cost-competitive without funding incentives.

### Methodology

The purpose of this analysis is to determine whether CHP technology needs funding incentives in order to be cost competitive. This analysis compares the cost of installing and using CHP technology without any funding incentives or offset capital costs and then determine if this cost is lower than the cost to simply purchase the electricity from the grid.

The domain of this analysis is U.S. natural gas fired electric power plants that adopt combined cycle gas turbine CHP technology. The Department of Energy estimates that 900 out of the next 1000 U.S. power plants will use natural gas. Furthermore, since a significant portion of CHP technologies are implemented in natural gas power plants, it is meaningful to conduct an analysis focused on CHP technologies in natural gas powered systems [40].

The resulting analysis takes two main steps: 1) First it calculates the levelized costs [41] to generate electricity when using CHP technology without any funding incentives or offset capital costs. 2) Then the levelized cost can be compared to the cost of electricity if purchased directly from the grid each year. If this cost is lower than the price of purchasing electricity directly from the grid, this would show that CHP technology is cost-competitive in combined cycle steam turbine applications in U.S. natural gas plants.

Further details of this methodology are presented in additional file 2.

### Results

#### Results of current cost evaluations

The results of this analysis show that the levelized costs for generating electricity with gas turbine powered CHP systems were between $0.05 and $0.06 (over the range of expected lifetimes) for generating electricity in our largest (83 MW) case, which remains lower than the projected yearly market price of electricity. In the smaller cases, however, the price to generate electricity using CHP systems remained higher than the annual market price of electricity: between $0.10 and $0.11 for our 5 MW case and between $0.33 and $0.36 in our 49 MW case.

For full results, refer to additional file 2.

### Discussion

We therefore conclude that in gas turbine applications CHP technology is cost-competitive without funding incentives in applications where generation capacity if average or higher than average (>83 MW). In applications where generating capacity is below average, CHP technology would need funding incentives to be cost effective.

Therefore, in cases of average or larger case applications of gas turbine CHP technology, the technology and market have progressed to a point at which there is no longer a need for funding incentives. This may help to explain our previous findings (in our policy analysis) that regulatory improvements do not explain the growth in adoption of CHP technologies because current regulations are focused primarily on large scale applications where there is no longer need for funding in order to make using CHP cost-competitive.

Large-scale applications are cost-competitive on their own, so funding incentives disproportionately skew the market towards large-scale applications, while small scale CHP applications show great potential for growth (as shown in the statistical analysis) but require funding to be cost-competitive. Furthermore, unnecessary funding may reduce the incentive to further develop efficiency of large-scale applications.

#### Opportunities for further analysis

The higher levelized cost for the smaller applications is most likely due to two main factors: 1) the higher incremental cost (S/kWh) of capital and installation for smaller cases and 2) the relatively fast overhaul of gas turbines.

The results of this analysis indicate a space for promising results in CHP applications with lower capital costs and turbines with longer life cycle.

A long life cycle allows costs to be levelized over a much longer time period because the capital costs only have to be incurred once per lifetime. A quick extension of the iterations used to calculate our levelized costs in the cast of gas turbines shows that an increase in life cycle lowers the cost of generating electricity below the market price of electricity. Lengthening the life cycle of gas turbines therefore holds the potential to dramatically reduce the cost to generate electricity and make CHP cost competitive even in smaller applications.

Research and development holds the primary potential to provide opportunities to lengthen gas turbine life cycles and reduce the incremental capital costs. In accomplishing these goals this will allow the technology and market to merge and increase the range of potential CHP applications in both large and small-scale sites.

Finally, though outside the scope of our analysis, steam turbines offer the benefits of a much-increased life cycle of up to 50 years with similar capital, fuel and O&M costs to those of gas turbines. In this way steam turbines already hold the potential for cost-competitiveness, especially in small-scale applications.

## Conclusions and recommendations

### Conclusions

Our cost-benefit analysis shows that large-scale natural gas turbine CHP systems are cost-competitive without financial incentives. This result is consistent with our policy analysis that suggests factor(s) other than state regulations explain the growth in CHP capacity. Because CHP growth during the ten year study period was concentrated in large-scale industrial facilities, the increase in capacity was likely due to the cost effectiveness of installing CHP systems, rather than the adoption of policies that favor CHP.

The analysis indicates that small-scale natural gas turbine CHP systems are not cost effective without financial incentives. Adopting the regulations examined in this paper would decrease the cost of CHP systems by removing barriers that are too costly for small-scale systems, suggesting that state policies may play a more important role in the future as the CHP industry trends towards smaller installations.

### Recommendations

The object of future efforts of state policy-makers to encourage the growth of Natural Gas powered CHP systems should be small-scale sites that are typically distributed generation facilities. Our cost-benefit analysis demonstrates that large-scale applications of natural gas turbines are cost-competitive without federal or state financial incentives. Our statistical analysis of state policies concludes that policy initiatives aimed at the general CHP marketplace are not effective at fostering growth of small-scale natural gas systems that are still facing cost and regulatory disadvantages.

Going forward, the majority of growth in the market share of CHP-generated electricity will come from distributed generation facilities producing less than 5MW. The most effective approach for growing this sector is for policy-makers and private-sector players to focus on removing the barriers uniquely facing small systems. We recommend four approaches that include: redirecting state and federal incentives, investing in research and development, directing regulations and policies that will quicken the adoption of Smart Grid technology, and focusing on outreach and education.

#### Direction of state and federal incentives

As shown in the synthesis of our analyses, we know that because large-scale applications of natural gas turbines are already cost-competitive, policy initiatives aimed at a CHP market dominated primarily by large-scale (and thus already cost-competitive) systems have not been effectively directed. Therefore, federal and state incentives should be directed towards ensuring that small-scale CHP applications are cost competitive.

In this re-direction, uniformity of policies is important; interstate coordination and cooperation to develop uniform policies could be important for further growth and will make it easier for firms to project future costs and benefits.

#### Research and development

Technology improvements are the most important driver for increasing small scale CHP growth. As shown in our Cost Benefit Analysis, increasing the life cycle of turbines and decreasing the incremental capital costs will decrease the levelized costs to generate electricity using CHP.

Therefore research and development of CHP technology holds the potential to make CHP technology cost-competitive in all size applications. Such developments would allow convergence of technology and the market and make funding incentives unnecessary. Furthermore, in a competitive market there will be more of an incentive to increase efficiency and further improve the effectiveness of CHP technology.

#### Smart grid

Improvements in U.S. electricity infrastructure are crucial to significantly improving the efficiency of the market. Currently regulating the energy grid presents a large set of unique challenges in coordinating between customers, utilities, state governments, and FERC. A common thread in these challenges is the lack of ability for all the essential players to communicate about electricity supply, demand, and prices. State and federal level investment in research, development and, most importantly, deployment of a Smart Grid would allow customers access to real-time pricing information of electricity. This would allow savings in energy efficiency to be more easily priced through in the marketplace, since customers would pay more for electricity that costs more to produce.

Small natural gas turbine CHP systems face challenges in connecting to the electricity grid because of the onerous and inconsistent state regulations we have detailed in this paper. One significant advantage to a smart grid is that grid operators are able to handle many more inputs of electricity. This would significantly decrease the barriers that small distributed generation facilities face by eliminating the built-in natural monopoly that currently facilitates the dominance of large centralized power plants.

Smart Grid technology will contribute to a vastly improved energy market. A market that values efficiency, facilitates information sharing, and eliminates barriers to entry will place an appropriate value on Combined Heat and Power systems.

#### Outreach and education

Finally, one of the remaining barriers to adoption of small-scale CHP use is that many users in the small-scale space (hospitals, schools, etc.) are unaware of the potential benefits of using CHP technologies in their facilities. Therefore education and outreach, by increasing awareness of the potential of CHP, would go great lengths to ensuring that viable CHP applications are not overlooked.

## List of abbreviations used

DOE: Department of Energy; DSIRE: Database of State Incentives for Renewables & Efficiency; EERE: DOE’s Office of Energy Efficiency and Renewable Energy; EIA: DOE’s Energy Information Administration; EPA: U.S. Environmental Protection Agency; FERC: Federal Energy Regulatory Commission; ICS: Interconnection Standards; IEEE: Institute of Electrical and Electronic Engineers; LHV: lower heating value; NREL: National Renewable Energy Laboratory; NSR: Clean Air Act’s New Source Review; OBR: Output Based Regulations; ORNL: Oak Ridge National Laboratory; PURPA: Public Utilities Regulatory Policy Act of 1978; QECB: Qualified Energy Conservation Bonds; RPS: Renewable Portfolio Standards.

## Competing interests

The authors declare that they have no competing interests.

## Supplementary Material

Additional file 1 Methodology for statistical analysis. Click here for file

Additional file 2 Methodology for cost benefit analysis. Click here for file
