# Supplementary material for: Combined heat and power systems: economic and policy barriers to growth
Source: Chem Cent J. 2012 Apr 23;6(Suppl 1):S3. doi: 10.1186/1752-153X-6-S1-S3 (PMC3332257; doi:10.1186/1752-153X-6-S1-S3)
Supplement: Additional file 1 — Methodology for statistical analysis. [file 1752-153X-6-S1-S3-S1.pdf]

Additional file 1: Methodology for statistical analysis

Dependent variable

The Dependent Variable was created with statistics from the Energy Information Administration's report, Electric Power Annual 2007 – State Data Tables [55]. The statistic showing the Nameplate Capacity of all sectors of electricity producers was compared to the Nameplate Capacity from only the CHP sector. We normalized the CHP capacity in MW by the total capacity in order to show the variable as a change in percentage of total state electricity production. We performed the same steps for the data from 1997 and measured the change in the percentage of total state capacity contributed by CHP systems over the ten-year period of the study.

Independent variables

Interconnection Standards (ICS)

In 2008, the EPA evaluated interconnection standards to determine which states had interconnection standards, and specifically, whether or not those standards favored distributed generation. Their assessment was based on the following criteria: standard interconnection forms, simplified procedure for smaller systems (>10 kW), timeline for application approval, system size limits, insurance requirements, and technical requirements. The categories used in the analysis for this paper are taken directly from the EPA's assessment. The values attached with each category and their meanings include:

0. Unfavorable: Policy in place, but has unfavorable attributes such as high fees and insurance requirements, or may only allow small systems to interconnect.

1. NoICS: No policy in place.

2. Neutral: Policy in place, but it doesn't favor DG specifically.

3. Favorable: Well defined policy with at least one beneficial attribute

Renewable Portfolio Standards (RPS)

Data came from the EPA, who has listed the states that have renewable portfolio standards and place, and specifically, which states include CHP or waste heat recovery systems as eligible technologies[56]. For the purposes of this paper, states were identified as either having an RPS that includes CHP or not:

0. No

1. Yes

Output based regulations (OBR)

Data came from the EPA, who lists the states that currently have output regulations [57]. For this paper's analysis, the variable identifies whether or not a state has adopted regulations:

0. No

1. Yes

Electricity restructuring (Restructure)

Data came from the EIA, who has identified which states have undergone electricity restructuring and has included a list of each state's process, outlining the progress states have made to date. The categories used in the analysis for this paper are taken directly from the EIA's listings [58]. The category and values attached with each include:

0. No

1. Suspended

2. Yes

Net metering (NetMet)

Data came from DSIRE and lists the states that include CHP as an eligible technology for net metering [59]:

0. No

1. Yes

Utility standby rates (Standby)

In 2008, the EPA assessed utility standby rates to identify which states have rates that value the costs and benefits of distributed generation. To measure this, the EPA reviewed the standby rates listed in utilities' rate schedules, called and questioned utilities with no standby rate listed in their rate schedule as to how they charge customers with on-site generation, and calculated hypothetical electric bills across each utility [60]. The EPA evaluated 91 utilities, the top two in each state. The categories used in the analysis for this paper are taken directly from the EPA's assessment. The values attached with each category and their meanings include:

0. No: Two utilities that only have conflicting rates or negative rates

1. Pending: States are considering effective policies

2. One: Effective policy for only one utility

3. Yes: Effective policy in place for top two utilities

Potential

ORNL has developed a list of states with the greatest technical potential for CHP adoption. The categories used in the analysis for this paper are taken directly from ORNL's assessment. The values attached with each category and their meanings include:

0. Potential is less than 1,000 MW

1. Potential between 1,000 - 3,000 MW

2.Potential between 3,000 - 8,000 MW

3. Potential is greater than 8,000 MW

Retail price of electricity (Price)

We created the continuous variable of the change in the retail price of electricity over the time horizon of the study from the Energy Information Administration's report, Electric Power Monthly-2009. The data is presented as the change in the average retail price, in cents per kWh, of electricity to ultimate customers of all end-use sectors from 1997 to 2007.

Regression data

State	Change in Share of Number of Producers	Change in Share of Generating Capacity	Stage of Active Restructuring	Renewable Portfolio Standards Include CHP	Status of Interconnection Standards	Presence of Output-Based Regulations	Presence of Net Metering	Presence of Utility Standby Rates	States with Greatest Potential	Change in Average Retail Price (c/KWH)
**STATE**	**PRODUCERS**	**CAPACITY**	**RESTRUCTURE**	**RPS**	**ICS**	**OBR**	**NETMET**	**STANDBY**	**POTENTIAL**	**PRICE**
AK	-5.0411	-6.4342	No	No	noICS	No	Yes	No	0	$3.21
AL	0.1698	1.4312	No	No	noICS	No	No	No	2	$2.25
AR	-6.7974	-0.2972	Suspend	No	unfavorable	No	Yes	No	1	$0.81
AZ	-6.5476	-0.4998	Suspend	Yes	neutral	No	Yes	No	1	$1.16
CA	-4.5629	-1.9127	Suspend	No	favorable	Yes	Yes	Yes	3	$3.26
CO	-5.7945	-2.7528	No	Yes	neutral	No	Yes	No	1	$1.81
CT	-3.7037	-2.2293	Yes	Yes	favorable	Yes	Yes	Yes	1	$5.93
DE	5.2381	26.4006	Yes	No	favorable	Yes	Yes	No	2	$4.35
FL	-8.1055	-1.8010	No	No	unfavorable	No	Yes	No	2	$3.14
GA	-11.9730	-2.1089	No	No	unfavorable	No	Yes	No	0	$1.49
HI	-13.6111	-3.2608	No	Yes	neutral	No	Yes	Pending	1	$8.80
IA	3.6281	4.9271	No	No	noICS	No	Yes	No	1	$0.87
ID	23.6086	29.6613	No	No	noICS	No	No	No	2	$1.17
IL	-19.3869	-0.1412	No	No	favorable	Yes	Yes	No	2	$0.77
IN	-5.2042	4.3689	Yes	No	noICS	No	Yes	No	1	$1.22
KS	15.0902	19.7176	No	No	noICS	No	Yes	No	2	$0.53
KY	63.0519	11.2061	No	No	unfavorable	No	Yes	No	2	$1.81
LA	-3.4607	6.8229	No	No	unfavorable	No	Yes	No	1	$2.41
MA	-7.6084	-2.2823	Yes	Yes	noICS	Yes	Yes	No	1	$4.71
MD	12.5000	1.6885	Yes	No	noICS	Yes	Yes	No	0	$4.52
ME	5.8891	-7.4907	Yes	No	noICS	Yes	Yes	One	2	$5.09
MI	-1.5297	-0.6541	Yes	Yes	favorable	No	Yes	No	1	$1.49
MN	-5.8987	-3.4619	No	No	noICS	No	No	No	1	$1.83
MO	-1.0462	0.2217	No	No	neutral	No	Yes	No	1	$0.47
MS	-5.2525	-2.4298	No	No	neutral	No	Yes	No	0	$2.12
MT	42.3986	9.2526	Suspend	No	noICS	No	Yes	No	2	$1.93
NC	-17.6441	-2.8688	No	Yes	neutral	No	Yes	No	0	$1.35
ND	5.9524	0.0569	No	Yes	noICS	No	Yes	No	1	$0.77
NE	-1.1765	-0.0418	No	No	noICS	No	Yes	No	0	$0.99
NH	-1.6393	-1.7940	Yes	No	favorable	Yes	Yes	No	1	$2.37
NJ	-12.1888	-2.0476	Yes	No	favorable	Yes	Yes	One	0	$2.47

State	Change in Share of Number of Producers	Change in Share of Generating Capacity	Stage of Active Restructuring	Renewable Portfolio Standards Include CHP	Status of Interconnection Standards	Presence of Output-Based Regulations	Presence of Net Metering	Presence of Utility Standby Rates	States with Greatest Potential	Change in Average Retail Price (c/KWH)
STATE	PRODUCERS	CAPACITY	RESTRUCTURE	RPS	ICS	OBR	NETMET	STANDBY	POTENTIAL	PRICE
NM	-14.9085	-1.2285	Suspend	No	noICS	No	Yes	No	0	$0.64
NV	19.0549	0.6926	Suspend	Yes	favorable	No	Yes	No	3	$4.38
NY	-8.4752	-4.5815	Yes	No	favorable	Yes	Yes	One	3	$4.09
OH	-12.4569	-0.3436	Yes	Yes	favorable	Yes	Yes	No	1	$1.66
OK	-4.3811	-2.5947	No	No	noICS	No	Yes	No	1	$1.87
OR	4.4721	3.4754	Suspend	No	favorable	No	Yes	One	3	$2.40
PA	-12.2128	0.3759	Yes	Yes	favorable	No	Yes	No	1	$1.09
RI	-16.0714	-7.1979	Yes	No	noICS	No	Yes	No	2	$2.44
SC	-2.7186	2.3744	No	No	neutral	No	No	No	0	$1.67
SD	4.1667	0.0640	No	Yes	noICS	No	No	No	2	$0.67
TN	7.8571	5.1309	No	No	noICS	No	No	No	3	$1.76
TX	-8.6556	3.6511	Yes	No	neutral	Yes	No	No	1	$3.95
UT	5.9821	1.9323	No	Yes	neutral	No	Yes	No	1	$1.24
VA	-19.7358	-5.2269	Suspend	No	neutral	No	Yes	No	0	$0.98
VT	0.4483	0.0800	No	No	favorable	No	Yes	No	1	$2.15
WA	-3.5088	-0.9600	No	Yes	favorable	No	Yes	No	2	$2.31
WI	2.7524	1.1807	No	No	neutral	No	Yes	No	1	$3.26
WV	-8.1594	-0.9019	No	No	noICS	No	Yes	No	0	$0.32
WY	-5.7143	0.6848	No	No	neutral	No	Yes	No	0	$0.96
