# Supplementary material for: Combined heat and power systems: economic and policy barriers to growth
Source: Chem Cent J. 2012 Apr 23;6(Suppl 1):S3. doi: 10.1186/1752-153X-6-S1-S3 (PMC3332257; doi:10.1186/1752-153X-6-S1-S3)
Supplement: Additional file 2 — Methodology for cost benefit analysis. [file 1752-153X-6-S1-S3-S2.pdf]

Additional file 2: Methodology for cost benefit analysis

Using levelized costs

Since one of the major costs is an up-front fixed capital cost (this includes installation costs), the initial cost to CHP users in the first year would be disproportionally larger than in any of the ensuing years so it would not be meaningful to compare the costs to the users of CHP technology each year with electricity prices in each year. For a more meaningful analysis, we calculate the *levelized costs* of generating electricity when using CHP technology. Levelized costs indicate the present value of the total cost of constructing and operating CHP technology in a natural gas plant over the technology's economic life, when converted to average annual payments [61].

In other words, it takes the main fixed costs (the initial capital costs, operations and maintenance and fuel costs) and assumes that the lump-sum costs can be spread out over time and shows what the equivalent yearly payment for the cost of installing a CHP plant would be. Although CHP systems produce a combination of heat and electricity, in this analysis we will assume that energy is converted to electricity. Therefore this assumes that if the user did not use CHP to produce the heat and electricity they would have to purchase the equivalent amount of electricity from the grid.

The calculation

Average lifetime levelized electricity generation cost (EGC) = $\Sigma[(C_t + O\&M_t + F_t - I_t)(1+r)^{-t}] / \Sigma[E_t(1+r)^{-t}]$. Where the variables relevant to calculating the average levelized cost of CHP are: C_t = Capital expenditures in the year *t*, $O\&M_t$ = Operations and maintenance expenditures in the year *t*, F_t= Fuel Expenditure in the year *t*, I_t= Funding Incentives in the year *t*, E_t = Electricity generation in the year *t*, r=Discount rate and *t*= year.

Basic assumptions

No offset capital costs, no avoided heat production costs.

Calculation assumptions

Discount rate

To determine the discount rate, we used a methodology based on rate of return to private investment. The Office of Management and Budget, a U.S. Government agency, states,

> "Base-Case Analysis. Constant-dollar benefit-cost analyses of proposed investments and regulations should report net present value and other outcomes determined using a real discount rate of 7 percent. This rate approximates the marginal pretax rate of return on an average investment in the private sector in recent years. Significant changes in this rate will be reflected in future updates of this Circular." [62]

Furthermore, the EPA cites project finance discount rates for various plants with a median value of 6.74 percent [63]. However, the current economic climate and the lack of availability of financing for capital-intensive projects make the base case somewhat unrealistic. For that reason, a minor adjustment is required to downsize the analysis, resulting in a reasonable increase of 1 percent to the discount rate, yield a final rate of 8 percent.

Electricity prices

2009 end-use price of electricity as sold to the Industrial sector in (2007 $ per kWh)

Electricity Price ($/kWh) (2009 price, 2007 $)	$0.07
Electricity Price Inflation Rate (%)	0.6%

[64]

Timeframe: three to six years

The timeframe for the analysis is based on the lifetime of the combined cycle gas turbine used in the examined CHP system. We want to determine the costs of purchasing, installing, and using CHP technology over the lifetime of the turbine assuming a one-time sunk cost for initial installation and capital costs In this analysis we assume that CHP plants run for every hour of the year, in reality they may run closer to 8,000 hours a year, but in order to determine the maximum potential of the turbine it is useful to consider the potential of continuous operation.

Most combined cycle gas turbines reliably last from 25,000 to 50,000 hours before requiring overhaul [65]. For rigor in our analysis, we examine the costs associated with both ends of this range. A 25,000-hour lifecycle is equivalent to approximately three years of continuous operation (8,760 hours) and a 50,000-hour lifecycle is equivalent to approximately 6 years of continuous operation.

This analysis calculated levelized costs in two timeframes, three years and six years, and assumes that the levelized cost would range between the two values depending on the lifecycle of the technology.

Operating assumptions

Overview:

Plant Capacity kW	5000	49000	83000
Efficiency	70%	73%	78%
**Electrical Output (kWh)**	105552099	810577429	1562101070
Operating Hours (per year)	8760	8760	8760
Fuel Input (MMBtu/hr)	56.315	451	780
Total Fuel Input (MMBtu)	493319	3950760	6832800
Fuel Input kWh	144591916	1157967756	2002693680

Nameplate capacity:

A scale comparison of 3 sizes, 5,000 kW, 49 MW, and 83 MW

In order to do a scale comparison of CHP plant costs, we calculate costs for CHP installations in average, median, and small sized natural gas plants. This analysis uses nameplate capacity as the determinant of the size of the plant and bases our other operating assumptions (fuel input, efficiency) on this capacity.

Nameplate capacity refers to amount in kW that a power plant can produce. Gas turbines can operate in plant sizes ranging from 500 kW to 250 MW. According to EIA data, the total nameplate capacity of the 5,494 natural gas plants in the United States is about 456,412 MW and the average capacity is 83 MW. The median capacity is 49 MW. Our analysis differentiates between funding for small and large scale CHP projects, classifying plants with nameplate capacities under the average

capacity of 83 MW as small-scale installations and those with nameplate capacities above 83 MW as large-scale applications.

The above data on operating assumptions based on nameplate capacity are used to estimate fuel input and efficiency for our chosen studies.

Fuel input

Identifying annual fuel input for each CHP application is necessary in order to determine fuel expenditures.

The data in the above table shows that the typical fuel input for a combined cycle gas turbine in a 5,000kW plant is about 56.3 MMBtu/hr and by plotting the other fuel input data for typical capacities of 1,000kW, 2,500kW, 5,000kW, 10,000kW, and 40,000kW and finding a line of best-fit we are able to estimate that typical fuel input for plant sizes of 49,000kW and 83,000kW were 451 MMBtu/hr and 780 MMBtu/hr respectively.

Typically, fuel input is measured in MMBtu/hr. To determine fuel input in kW we used our estimate of operating hours to convert to MMBtu and the conversion 1 MMBtu= 293.1 kWh to convert to kWh.

Efficiency

Derived technical characteristics [66]

Electrical Capacity (kW)	1,000	2,500	5,000	10,000	40,000
Fuel Input (MMBtu/hr)	14.217	32.83	56.315	105.15	389.943

Steam Output	6.54	14.451	22.361	44.743	175.474
Steam Output/Fuel Input	46.0%	44.0%	39.7%	42.6%	45.0%
Power Steam Ratio	0.522	0.59	0.763	0.763	0.778
Net Heat Rate	6042	5907	5673	4922	4265
Thermal Output as Fraction of Fuel Input	0.46	0.44	0.40	0.43	0.45
Electric Output as Fraction of Fuel Input	0.24	0.26	0.3	0.32	0.35
Overall Efficiency (%)	70.0%	70.0%	70.0%	75.0%	80.0%

CHP plant efficiency is determined as the combine thermal and electric output as a fraction of fuel input. Thermal output is determined by the steam output over fuel input and electric output is determine by using the power to steam ratio and the steam output over fuel input to calculate electric output as a fraction of fuel cost.

Given these calculations and the data available for typical plant sizes (refer to above table) we used the same method as when we estimated fuel input for larger plant sizes to estimate a 73 percent and 78 percent efficiency for plant capacities of 49 MW and 83 MW respectively.

Costs

Conversions and related calculations: 1 cubic foot= 1080-1034 Btu, 1000 cubic feet=1057000 Btu, 1 kWh=3413 Btu, 1 MMBtu= 293.1 kWh, 1000 cubic feet=309.7 kWh, Electrical Output= % efficiency * Btu/hr input.

Plant capital costs

Plant Capacity kW	5000	49000	83000
Efficiency	73%	70%	78%
**Electrical Output (kWh)**	105552098.8	810577429.2	1562101070
Operating Hours per year	8760	8760	8760
Capital Cost $/kW	$1,419.18	$1,054.44	909.4529527
Capital Cost ($/kWh)	$0.1620	$0.1204	$0.1038
**Capital Expenditures ($)**	$17,100,200.4663	$97,568,928.1208	$162,175,505.8181

O&M costs

Plant Capacity kW	5000	49000	83000
Efficiency	73%	70%	78%
**Electrical Output (kWh)**	105552098.8	810577429.2	1562101070
Incremental O&M Costs in $/kWh	$0.2211	$0.0050	$0.0050
**O&M Expenditures ($)**	$23,337,661.21	$4,059,843.10	$7,823,910.49

To determine plant capital and operations and maintenance costs for our test sizes we used data for gas turbine capacities of 1 MW, 5 MW, 10 MW, 20 MW, and 40 MW and applied the same method as used for estimating efficiency and fuel input in larger plant sizes.

Fuel costs

Fuel Price (2007 $ per thousand cubic ft)	$6.81
Fuel Price ($/kWh)[67]	$0.02

[68]

Plant Capacity kW	5000	49000	83000
Efficiency	73%	70%	78%
**Electrical Output (kWh)**	105552098.8	810577429.2	1562101070
Operating Hours per year	8760	8760	8760
Fuel Input (MMBtu/hr)	56.315	451	780
Total Fuel Input (MMBtu)	493319.4	3950760	6832800
Fuel Input kWh	144591916.1	1157967756	2002693680
Fuel Costs ($/kWh)	$0.0220	$0.0220	$0.0220
**Fuel Expenditures ($)**	$3,178,062.80	$25,451,590.54	$44,018,271.88

To determine expenditures we multiplied electrical output (measured in kWh) by incremental costs (in $/kWh) to get expenditures. Electrical Output = % efficiency * Btu/hr Input.

Results of cost benefit analysis

Basic cost of CHP plant

Plant Capacity kW	5000	49000	83000
Efficiency	70%	73%	78%
Electrical Output (kWh)	105552098.8	810577429.2	1562101070
Operating Hours per year	8760	8760	8760
Capital Cost $/kW	$1,419.18	$1,054.44	909.4529527
Capital Cost ($/kWh)	$0.1620	$0.1204	$0.1038
**Capital Expenditures ($)**	$17,100,200.4663	$97,568,928.1208	$162,175,505.8181
Fuel Input (MMBtu/hr)	56.315	451	780
Total Fuel Input (MMBtu)	493319.4	3950760	6832800
Fuel Input kWh	144591916.1	1157967756	2002693680
Fuel Costs ($/kWh)	$0.0220	$0.0220	$0.0220
**Fuel Expenditures ($)**	$3,178,062.80	$25,451,590.54	$44,018,271.88
Incremental O&M Costs in $/kWh	$0.2211	$0.0050	$0.0050
**O&M Expenditures ($)**	$23,337,661.21	$4,059,843.10	$7,823,910.49
Total CHP plant Costs ($/kWh)	$0.4051	$0.1474	$0.1308
Total CHP plant expenditures	$43,615,924.4701	$127,080,361.7569	$214,017,688.1895

Average lifetime levelized electricity generation costs (EGC)

Σ CHP Plant Costs over lifetime (3 years)	$205,134,981.64	$162,903,066.40	$799,344,232.51
Σ CHP Plant Costs over lifetime (6 years)	$326,848,732.55	$273,799,617.35	$1,343,499,234.37
Σ kW Electricity Generated over lifetime (3 years)	$3,475,715,791.23	$452,602,161.52	$6,698,211,870.07
Σ kW Electricity Generated over lifetime (6 years)	$6,346,396,160.46	$826,417,576.30	$12,230,432,131.85
**EGC over lifetime (3 years)**	**$0.1193**	**$0.3599**	**$0.0590**
**EGC over lifetime (6 years)**	**$0.1098**	**$0.3313**	**$0.0515**
